# Generation of Liposomes to Study the Effect of *Mycobacterium Tuberculosis* Lipids on HIV-1 *cis*- and *trans*-Infections

**DOI:** 10.3390/ijms22041945

**Published:** 2021-02-16

**Authors:** Marion Pouget, Anna K. Coussens, Alessandra Ruggiero, Anastasia Koch, Jordan Thomas, Gurdyal S. Besra, Robert J. Wilkinson, Apoorva Bhatt, Georgios Pollakis, William A. Paxton

**Affiliations:** 1 Department of Clinical Infection, Microbiology and Immunology, Institute of Infection, Veterinary and Ecological Sciences, University of Liverpool, Liverpool L69 7BE, UK; marion.pouget@ucd.ie (M.P.); aler@liverpool.ac.uk (A.R.); hljthom2@liverpool.ac.uk (J.T.); 2 UCD Centre for Experimental Pathogen Host Research, University College Dublin, Belfield, Dublin 4, Ireland; 3 Wellcome Center for Infectious Diseases Research in Africa, Institute of Infectious Disease and Molecular Medicine and Department of Medicine, University of Cape Town, Observatory, Cape Town 7925, South Africa; coussens.a@wehi.edu.au (A.K.C.); a.koch@uct.ac.za (A.K.); robert.wilkinson@uct.ac.za (R.J.W.); 4 Walter and Eliza Hall Institute of Medical Research, Parkville 3279, Australia; 5 Academic Department of Pediatrics (DPUO), IRCCS Ospedale Pediatrico Bambino Gesù, Piazza S. Onofrio 4, 00165 Rome, Italy; 6 Institute of Microbiology and Infection and School of Biosciences, University of Birmingham, Birmingham B15 2TT, UK; g.besra@bham.ac.uk (G.S.B.); a.bhatt@bham.ac.uk (A.B.); 7 Department of Infectious Diseases, Imperial College, London W2 1PG, UK; 8 The Francis Crick Institute, London NW1 1AT, UK

**Keywords:** HIV-1, TB, *Mycobacterium tuberculosis*, in vitro, DC-SIGN, liposomes, *trans*-infection, SL1, TDM, PDIM, H37Rv, HN878, CDC1551, EU127, BCG, *M. smegmatis*

## Abstract

Tuberculosis (TB) is the leading cause of death among HIV-1-infected individuals and *Mycobacterium tuberculosis* (*Mtb*) co-infection is an early precipitate to AIDS. We aimed to determine whether *Mtb* strains differentially modulate cellular susceptibility to HIV-1 infection (*cis*- and *trans*-infection), via surface receptor interaction by their cell envelope lipids. Total lipids from pathogenic (lineage 4 *Mtb* H37Rv, CDC1551 and lineage 2 *Mtb* HN878, EU127) and non-pathogenic (*Mycobacterium bovis* BCG and *Mycobacterium smegmatis*) *Mycobacterium* strains were integrated into liposomes mimicking the lipid distribution and antigen accessibility of the mycobacterial cell wall. The resulting liposomes were tested for modulating in vitro HIV-1 *cis*- and *trans*-infection of TZM-bl cells using single-cycle infectious virus particles. *Mtb* glycolipids did not affect HIV-1 direct infection however, *trans*-infection of both R5 and X4 tropic HIV-1 strains were impaired in the presence of glycolipids from *M. bovis*, *Mtb* H37Rv and *Mtb* EU127 strains when using Raji-DC-SIGN cells or immature and mature dendritic cells (DCs) to capture virus. SL1, PDIM and TDM lipids were identified to be involved in DC-SIGN recognition and impairment of HIV-1 *trans*-infection. These findings indicate that variant strains of *Mtb* have differential effect on HIV-1 *trans*-infection with the potential to influence HIV-1 disease course in co-infected individuals.

## 1. Introduction

Human immunodeficiency virus type 1 (HIV-1) and *Mycobacterium tuberculosis* (*Mtb*) co-infection results in a loss of numerous immunological functions and ultimately leads to death when not treated. The WHO estimated in 2019 that 208,000 people died of HIV-associated tuberculosis [1]. Both, *Mtb* and HIV-1 can induce profound changes in the host immune response resulting in increased rates of active *Mtb* disease or the exacerbation of HIV-1 infection. *Mtb* creates a favorable environment for HIV-1 direct infection (*cis*-infection) and/or replication, as *Mtb* infection leads to the activation and induction of immune responses [2,3,4].

Due to the glycosylation of the mycobacterial cell wall and HIV-1 envelope protein gp120, both pathogens engage C-type lectin receptors [5]. In the case of HIV-1, numerous cells of the immune system, including dendritic cells, can bind and capture HIV-1 via an array of C-type lectins expressed on the cell surface. Binding not only promotes the capture, processing and subsequent presentation of HIV-1 antigen to other cells of the immune system but can result in the capture and transfer of HIV-1 virions to susceptible cells, a mechanism termed *trans*-infection [6,7,8,9]. Different receptors have been described to be involved with *trans*-infection, such as the mannose receptor MR [6,10,11] and the C-type lectin receptor DC-SIGN [12,13,14,15], alongside Siglec via interactions with ganglioside GM3 localised on the virus membrane [16,17,18,19,20].

The mycobacterial cell envelope is complex and composed of carbohydrate polymers such as arabinogalactan (AG) and distinct lipids including mycolic acids (MA) linked to peptidoglycans (PG) and free lipids trehalose dimycolate (TDM), phthiocerol dimycocerosate (PDIM/DIM), sulfolipids (SLs), phosphatidylinositol mannosides (PIM), lipomannan (LM) and lipoarabinomannan (LAM) [21,22,23,24,25,26]. These outer envelope lipids play an important role in structural integrity and can modulate the host immune response, therefore influencing *Mycobacterium* pathogenicity. MA constitutes a natural barrier of the bacteria and the nature of its composition and oxygenation can influence *Mtb* pathogenicity [27,28] by modulation of macrophage activation and differentiation [29,30]. In addition, the inflammatory response can be associated with AG and PG structures [31]. TDM (also known as cord factor) is a major contributor to inflammation [32,33,34,35] and can also modulate macrophage activation [32,36,37], macrophage surface markers expression [38] and granuloma formation [39,40]. SLs are sulphated trehalose esters and several studies using *Mtb* mutants in SL1 biosynthesis have revealed its role in *Mtb* virulence, for example, mmpL8 knock-out mutants of *Mtb* have been shown to attenuate bacterial virulence in mouse models [41,42]. PDIMs have been described to play a role in *Mtb* virulence [43,44] and the presence of PDIMs at the bacterial surface can aide escape from macrophage recognition [45,46], mask pathogen associated molecular patterns (PAMPs) [47], and prevent macrophage recruitment [48]. PIMs are glycoconjugates and represent the major components of the mycobacterial cell envelope and are precursors of LM, LAM and mannan capped lipoarabinomannan (ManLAM). Mannoside caps associated to PIMs can be recognised by phagocytic receptors such as C-type lectins, including MR and DC-SIGN [49,50,51]. Additionally, LM has been described as a TLR2 and TLR4 ligand, modulating anti- and pro-inflammatory responses against the bacteria [52,53,54,55,56].

The proportion of lipids present within the mycobacterial cell wall can vary between *Mycobacterium* strains resulting in different biochemical properties [57,58]. Variation in lipid compositions inside *Mtb* species can impact on their pathogenicity. Different clinical *Mtb* strains are associated with important disparities in immune response activation such as CDC1551 and HN878 [59,60,61,62]. Compared to the lineage 4 CDC1551, the lineage 2 Beijing strain HN878 induces an altered immune response partly due to its expression of phenolic glycolipids (PGL), increasing its virulence [63,64,65,66]. In order to measure and compare the impact of glycolipids derived from different *Mycobacterium* strains on HIV-1 *cis*- and *trans*-infection, we developed liposomes which contained *Mycobacterium* total lipid extracts from lineage 4 *Mtb* H37Rv, and CDC1551, lineage 2 *Mtb* HN878 and non-pathogenic *M. bovis* BCG and *M. smegmatis*. These liposomes are artificial vesicles composed of a mix of phosphatidylcholine (PC) and cholesterol (Ch) and used as the experimental foundation in our assays. In many cases, liposomes have been used as a carrier for various applications in pharmacology, including for diagnostic purposes, therapeutic treatments of cancer and for use in vaccine technologies [67]. In the context of TB, the interest in using liposomes for vaccination strategies is important and well described in the literature [68,69,70,71,72,73].

## 2. Results

### 2.1. Production of Liposomes Containing Mycobacterium Total Lipids

**Figure 1:**
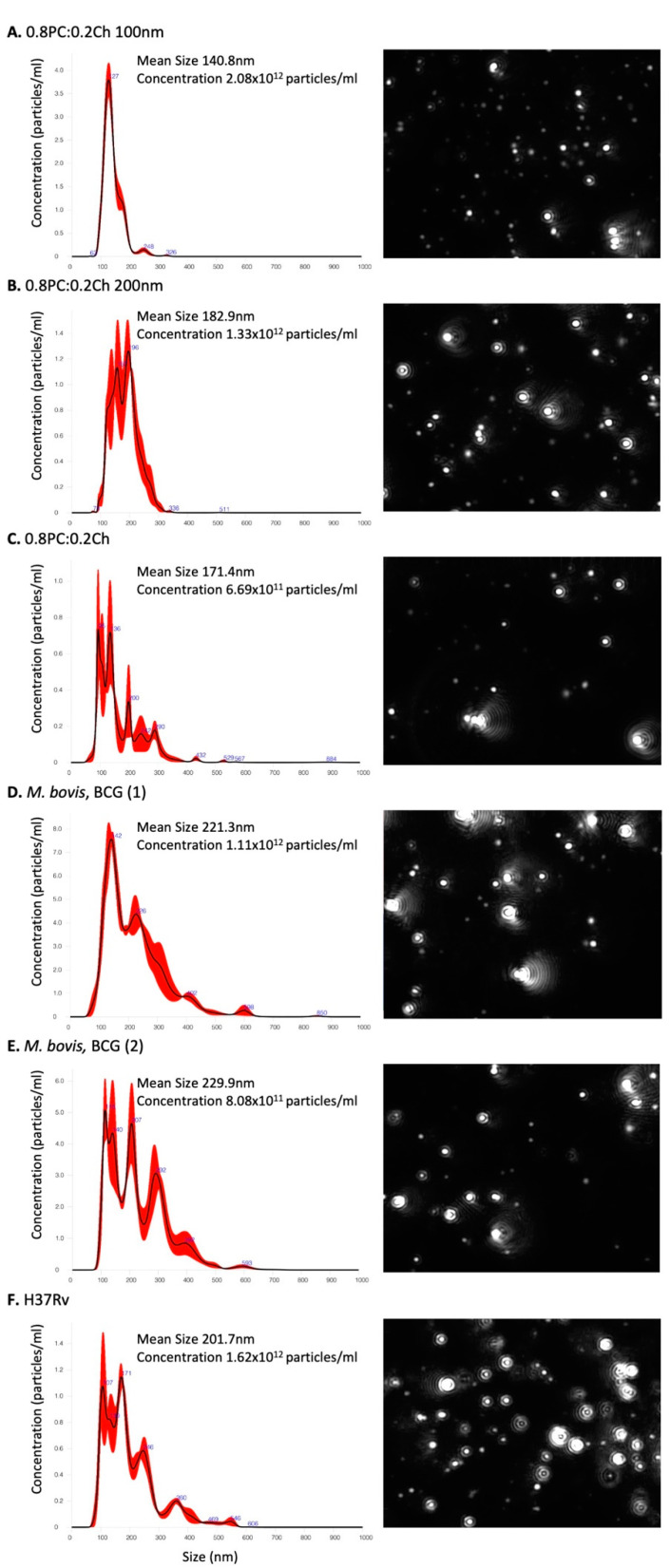
Liposome-size analysis by NanoSight particle tracking. (**A**) Liposomes generated with the Extruder system at 100 nm size, (**B**) Liposomes generated with the Extruder system at 200 nm size (**C**) 0.8PC:0.2Ch, (**D**) BCG batch n° 1, (**E**) BCG batch n° 2 and (**F**) H37Rv liposomes made by sonication without the Extruder step. Liposome suspensions were diluted 1:1000–1:2000 in PBS, and three 60 s videos were recorded. The data shown are from one experiment. On the right are graphs showing the results of particle concentration and their size measurement; and on the left are liposomes observed at the screen shot from NTA video.

**Figure 2:**
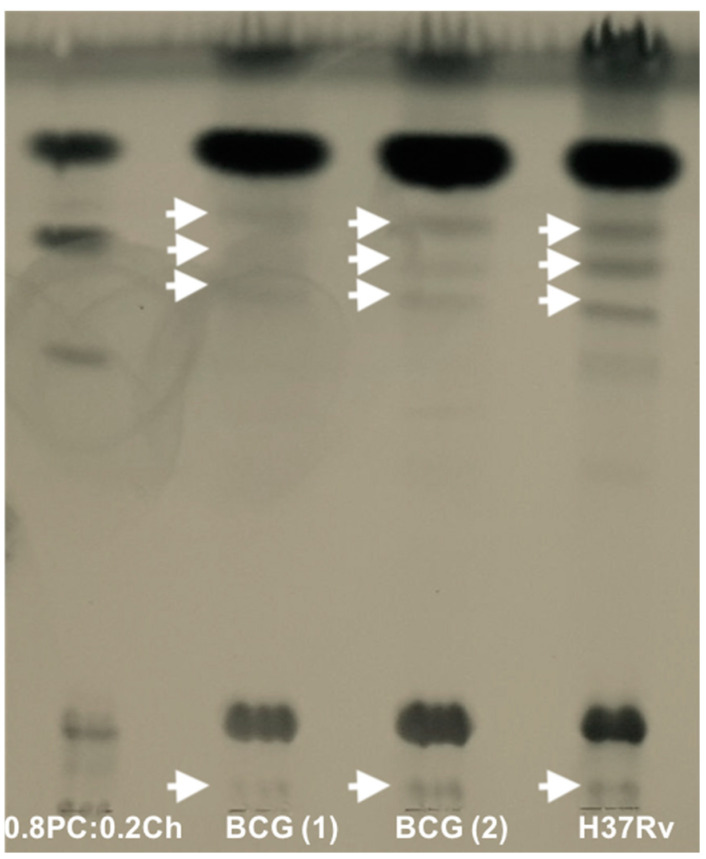
Thin-layer chromatography (TLC) analyses of 0.8PC:0.2Ch, BCG and H37Rv liposomes. 10 µL of liposomes solution made in water were spotted and dried on a silica gel 60 F254 plate. Separation occurred in 60:16:2 CHCl_3_:MeOH:H_2_O solvent and was visualised by staining with molybdophosphoric acid and charring. The arrows show the presence of lipids from mycobacterial origin. The data are from one representative experiment.

The initial aim was to identify whether we could incorporate lipid extract antigens from variant *Mycobacterium* strains into liposomes. Unilamellar liposomes were generated with total lipid extracts from differing strains as described. We characterised 0.8PC:0.2Ch, *M. bovis* and *Mtb* H37Rv liposomes generated by NanoSight technology (Figure 1) and Thin-layer chromatography TLC (Figure 2).

Visualisation of liposome particles using NanoSight NS300 optics and software was used to define their size and distribution (Figure 1). Calibrated liposomes were generated with an additional step of extrusion following sonication, producing: 0.8PC:0.2Ch liposomes at 100 nm (Figure 1A) and 200 nm (Figure 1B), respectively. In parallel, 0.8PC:0.2Ch liposomes were produced with a step of sonication only with no calibration (Figure 1C). Two separate batches of *M. bovis* liposomes were generated (Figure 1D,E) and each measured on average 221.3 and 229 nm, respectively, while the mean size of H37Rv liposomes (Figure 1F) was 201.7 nm. The similar sizes and distribution of the two independent preparations of *M. bovis* liposomes demonstrates the reproducibility of the liposome generation technique. Due to a single sonication step, the population of *M. bovis* and H37Rv liposomes generated is heterogenous compared to calibrated liposomes at 100 nm and 200 nm. Additionally, *Mycobacterium* liposomes appear larger than the calibrated liposomes [0.8PC:0.2Ch 100 nm (140.8 nm), 0.8PC:0.2Ch 200 nm (182.9 nm)] and 0.8PC:0.2Ch liposomes generated with a single sonication step (171.4 nm, Figure 1C). This size variation is likely explained by the incorporation of the *Mycobacterium* lipids onto *M. bovis* and H37Rv liposomes. When comparing particle concentrations, we observed similar order values between liposome preparations included between 0.6 − 2 × 10^12^ particles per ml. A TLC analysis of lipids extracted from the liposomes revealed two sets of lipid species. The first set was found in all preparations including the ‘empty’ liposomes and were thus liposome derived (PC and Ch, shown). The other set of lipid species (shown by arrows) were of relatively lower proportions and were only found in liposomes that were reconstituted with mycobacterial lipids and thus were of mycobacterial origin (Figure 2). However, between *M. bovis* and H37Rv we observed variation in the intensity of the lipid bands, suggesting differences in lipid proportions contained in the liposomes. The analysis, therefore, demonstrates the integration of specific *Mycobacterium* lipids into liposomes.

### 2.2. Mycobacterium Lipids Do Not Affect HIV-1 Cis-Infection

In order to measure the direct impact of *Mtb* liposomes on HIV-1 *cis*-infection (direct infection), a non-productive HIV-1 pseudo-typed viral particle system (single round of infection) was used in vitro. This system facilitates investigation into interaction or interference of HIV-1 entry and infection via the co-receptors CCR5 and CXCR4 in the presence of *Mycobacterium* derived liposomes. To this end, two types of HIV-1 pseudo-typed viruses were produced, expressing either HIV-1 LAI envelope (HIV-1 X4, utilisng CXCR4) or HIV-1 BAL envelope (HIV-1 R5, utilising CCR5).

The impact of 0.8PC:0.2Ch liposomes (containing PC and cholesterol only) on the viability of the target cells used in this in vitro system (TZM-bl) were tested (Figure S1). Control liposomes composed of a mix of PC and Ch did not affect TZM-bl viability after 48 h of incubation. Additionally, we observed that 0.8PC:0.2Ch liposomes did not interfere with HIV-1 *cis*-infection compared to other liposomes compositions (Figure S2). For these reasons, liposome produced in the ratio 0.6PC:0.2Ch:0.2*Mycobacterium* were chosen to build liposomes incorporating *Mycobacterium* total lipid extracts.

**Figure 3:**
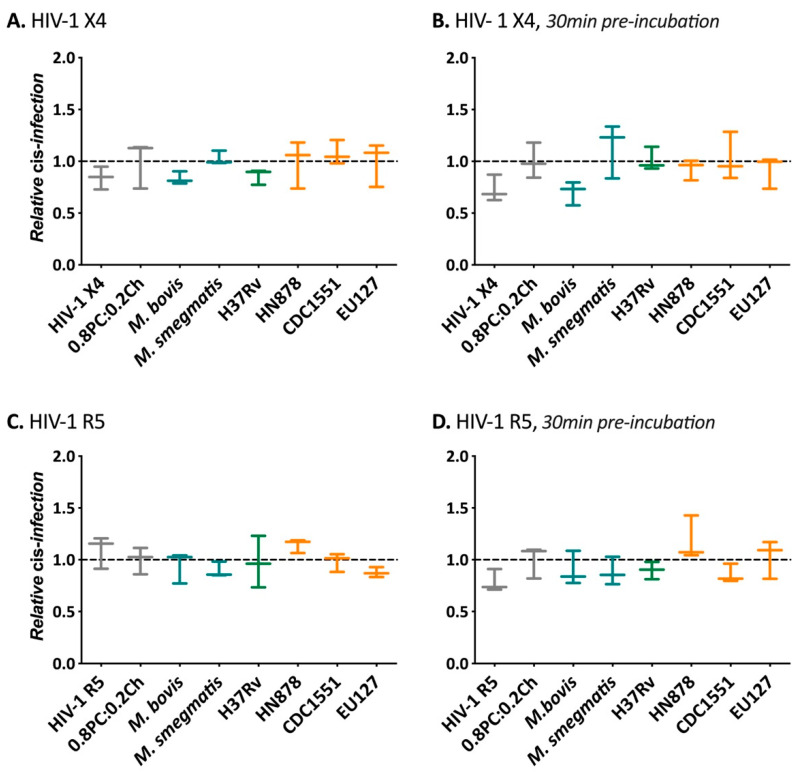
Influence of *Mycobacterium* liposomes on HIV-1 *cis*-infection. Cells were infected with 8 ng CA-p24 of (**A**,**B**) pSG3-LAI (HIV-1 X4), (**C**,**D**) pSG3-BAL (HIV-1 R5) and pSG3Δenv (ΔpSG3) where (**A**,**C**) virus input with 100 ng of liposomes at the same time or (**B**,**D**) 100 ng of liposomes added to TZM-bl cells 30 min prior to adding virus. 48 h after infection cells were lysed to measure luciferase activity (relative light unit, RLU). The RLUs produced for each experiment were normalised to the average value of the negative control 0.8PC:0.2Ch liposomes. 0.8PC:0.2Ch is used here as a negative control and reference. For the data shown, *n* = 3. Mann–Whitney unpaired t-test was performed for (**A**–**D**).

The influence of *Mycobacterium* liposomes containing *M. bovis*, *M. smegmatis*, *Mtb* H37Rv, HN878, CDC1551 and EU127 glycolipids was then assessed for modulating *cis*-infection of TZM-bl cells using HIV-1 X4 and HIV-1 R5 viruses (Figure 3). Two conditions were tested, where liposomes were added to cells at the same time as virus and where liposomes were incubated with cells 30 min prior to infection. In order to compare results from separate experiments, the raw relative light units (RLU) produced on every plate were normalised to the average value of the negative control. For both viruses, in comparison with TZM-bl *cis*-infection in the presence of 0.2PC:0.8Ch liposomes, we did not observe any significant impact of *Mtb* liposomes under the two conditions, suggesting that the tested glycolipids did not have the capacity to interfere with HIV-1 cell attachment and cell entry via co-receptor recognition. Our results indicate that the derived liposomes described do not alter HIV-1 direct infection of TZM-bl cells.

### 2.3. M. bovis, Mtb H37Rv and EU127 Lipids Interfere with HIV-1 Trans-Infection Mediated by DC-SIGN

**Figure 4:**
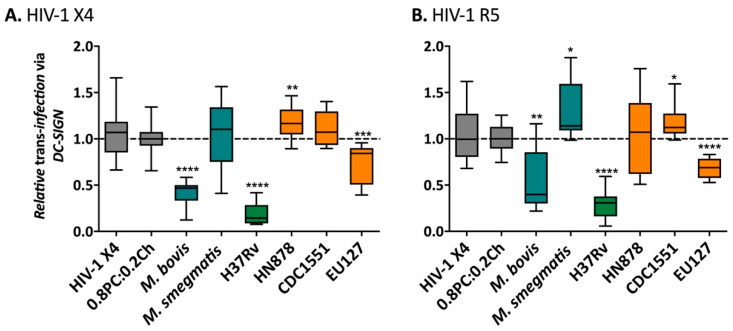
Influence of the presence of *Mycobacterium* liposomes on HIV-1 *trans*-infection via Raji-DC-SIGN cells. (**A**) 12.5 ng CA-p24 pSG3-LAI (HIV-1 X4) or (**B**) 20 ng CA-p24 pSG3-BAL (HIV-1 R5). After capture the cells were washed and co-cultured with TZM-bl cells. The luciferase activity (RLU) was read after 48 h. The RLUs produced for each experiment were normalised to the average value of the negative control 0.8PC:0.2Ch liposomes. The data shown are a pool of at least two independent experiments where *n* ≥ 3 in total. Mann–Whitney unpaired *t*-test was performed for (**A**,**B**) and *p*- value represented with * for *p*-value < 0.05, ** for *p*-value < 0.01, *** for *p*-value < 0.001, **** for *p*-value < 0.0001.

We next investigated the influence of *Mtb* lipids on HIV-1 *trans*-infection via binding the DC-SIGN receptor using the same in vitro system of pseudo-typed virus particles that allows a single-round of infection. In this assay, Raji-DC-SIGN cell lines were used to support capture-transfer of HIV-1 R5 or X4 virions due to their expression of DC-SIGN receptor and inability to be infected by the virus through lack of CD4 receptor expression. *Mycobacterial* liposomes were pre-incubated with Raji-DC-SIGN before HIV-1 X4 (Figure 4A) and HIV-1 R5 (Figure 4B) capture. The efficacy of capture-transfer of HIV-1 was normalised to the value of *trans*-infection in the presence of liposomes 0.8PC:0.2Ch (negative control). We observed a strong inhibition of the efficacy of HIV-1 X4 and R5 *trans*-infection mediated by DC-SIGN receptor in the presence of *M. bovis*, *Mtb* H37Rv and EU127 liposomes. Indeed, the non-pathogenic mycobacterial strain *M. bovis* inhibited HIV-1 capture-transfer by 2.4-fold for HIV-1 X4 (*p*-value < 0.0001) and 1.9-fold for HIV-1 R5 (*p*-value = 0.0050). The *Mtb* pathogenic strain, H37Rv, strongly blocked *trans*-infection of HIV-1 X4 and R5 virus by 5.2- and 3.4-fold respectively (*p*-value < 0.0001), and EU127 by 1.4-fold for HIV-1 X4 (*p*-value = 0.0009) and 1.5-fold for HIV-1 R5 (*p*-value < 0.0001).

Conversely, only small and variable effects were observed for *M. smegmatis*, *Mtb* HN878 and CDC1551. *Mtb* HN878 increased *trans*-infection efficacy of HIV X4 by 1.2-fold (*p*-value = 0.0052), whilst there was no effect on X4 *trans*-infection by *M. smegmatis* or *Mtb* CDC1551 liposomes. Conversely, HIV-1 R5 *trans*-infection exhibited the opposite effect, whereby HN878 liposomes did not affect capture-transfer but *M. smegmatis* and *Mtb* CDC1551 liposomes increased the efficacy of R5 by 1.3-fold (*p*-value = 0.0190) and 1.2-fold (*p*-value = 0.0414), respectively.

The impact of *M. smegmatis*, *Mtb* HN878 and CDC1551 total lipids on HIV-1 X4 and R5 *trans*-infection via DC-SIGN was minimal compared to the effect *M. bovis*, *Mtb* H37Rv and EU127, suggesting that glycolipids from these three strains could bind DC-SIGN thereby blocking virus *trans*-infection of the target cells.

### 2.4. Implication of SL1, PDIM and TDM Lipids in HIV-1 Trans-Infection Mediated by DC-SIGN

**Figure 5:**
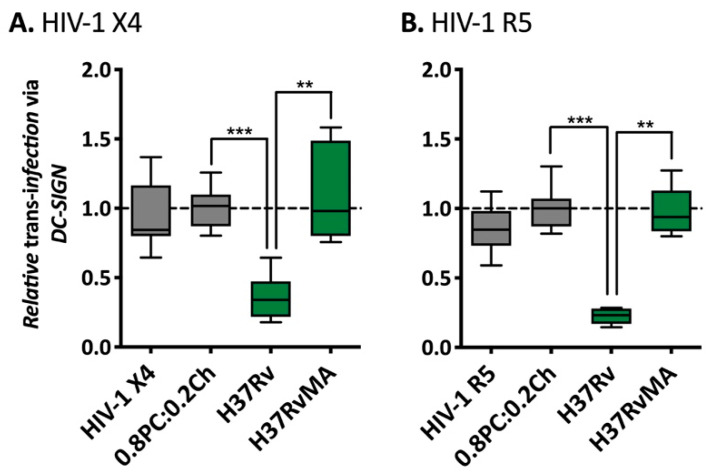
Comparison of the influence different H37Rv on HIV-1 *trans*-infection via Raji-DC-SIGN cells. (**A**) 12.5 ng CA-p24 pSG3-LAI (HIV-1 X4) or (**B**) 20 ng CA-p24 pSG3-BAL (HIV-1 R5). After capture the cells were washed and co-cultured with TZM-bl cells. The luciferase activity was read after 48 h. The RLUs produced for each experiment were normalised to the average value of the negative control 0.8PC:0.2Ch liposomes. The data shown are a pool of at least two independent experiments where *n* ≥ 6 in total. Mann–Whitney unpaired t-test was performed for (**A**,**B**) and *p*-value represented with * for *p*-value < 0.05, ** for *p*-value < 0.01, *** for *p*-value < 0.001, **** for *p*-value < 0.0001.

To further investigate the observed disruption of HIV-1 *trans*-infection by *Mtb* H37Rv glycolipids associated into liposomes, we compared the influence of total lipids from a variant H37Rv strain obtained from a different lab: H37RvMA (Figure 5) where the two lineages are differing in the number of local passage, inducing genotypic variation [74]. Raji-DC-SIGN were pre-incubated with liposomes prior to HIV-1 X4 (Figure 5A) or HIV-1 R5 (Figure 5B) capture-transfer to TZM-bl cells. Interestingly, while the presence of H37RvAE liposomes decreased HIV-1 X4 and R5 *trans*-infection via DC-SIGN to 0.35 (*p*-value = 0.0004) and 0.22 efficacy (*p*-value = 0.0004), H37RvMA did not affect virus capture-transfer with a mean efficacy of 1.09 (HIV-1 X4) and 0.98 (HIV-1 R5). Specific mutations are observed in H37RvAE compared to H37RvMA, including mutation in *ppsA* that encodes a multidomain polyketide synthase involved in PDIM biosynthesis [74]. While mouse passaged H37Rv strains do produce PDIM, it is likely that variations in PDIM levels occur in H37RvAE and H37RvMA liposomes, interfering with virus DC-SIGN receptor recognition. Given the low sensitivity of the TLC method in detecting the incorporated mycobacterial lipids (Figure 2), it is difficult to quantify this difference in PDIM levels in liposomes derived from the lipids of the two H37Rv strains.

**Figure 6:**
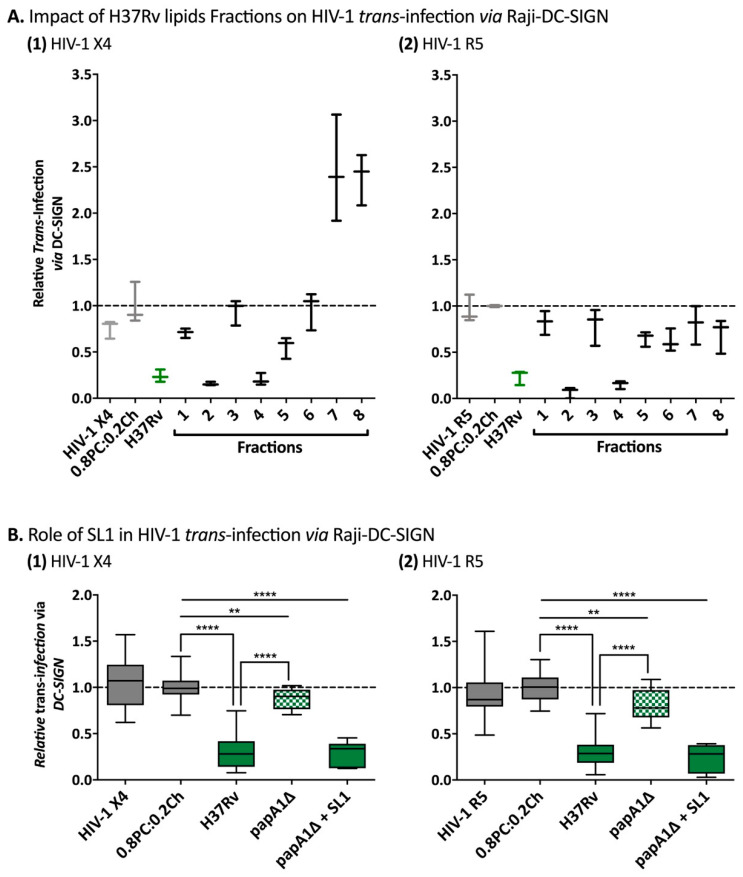
Role of H37Rv glycolipids in HIV-1 *trans*-infection via DC-SIGN. (**A**) H37Rv fractions 1 to 8 have been integrated into liposomes following the ratio 0.6PC:0.2Ch:0.2fraction and were tested on HIV-1 *trans*-infection via DC-SIGN in parallel of 0.8PC:0.2Ch and H37Rv liposomes. The data shown are from one experiment, *n* = 3. (**B**) H37Rv papA1Δ and H37Rv papA1Δ + SL1 were tested in parallel of 0.8PC:0.2Ch and H37Rv liposomes on HIV-1 *trans*-infection. The data shown are a pool of at least two independent experiments where *n* ≥ 6 in total. For (**A**,**B**) 0.5 × 10^6^ Raji DC-SIGN were pre-incubated during 30 min with 100 μg liposomes or 50 µL of media. The cells were then incubated for 2 h with HIV-1 pseudo-typed virus (1) 12.5 ng CA-p24 pSG3-LAI (HIV-1 X4) or (2) 20 ng CA-p24 pSG3-BAL (HIV-1 R5). After capture the cells were washed and co-cultured with TZM-bl cells. The luciferase activity was read after 48 h. The RLUs produced for each experiment were normalised to the average value of the negative control 0.8PC:0.2Ch liposomes. Mann–Whitney unpaired t-test was performed for (**A**,**B**) and *p*-value represented with * for *p*-value < 0.05, ** for *p*-value < 0.01, *** for *p*-value < 0.001, **** for *p*-value < 0.0001.

In order to identify glycolipids from *Mtb* H37Rv that influence HIV-1 *trans*-infection via DC-SIGN, total lipids from H37Rv were fractionated and the fractions obtained were associated into liposomes to assess their impact on HIV-1 *trans*-infection (Figure 6A). For both virus tropisms, we observed inhibition of virus capture-transfer via DC-SIGN with liposomes derived from fractions 2 and 4 by 6.4 and 5-fold for HIV-1 X4 and 14.5 and 6.6-fold for HIV-1 R5, respectively. Fractions 1 and 5 liposomes also inhibited virus *trans*-infection but with a weaker effect with a decrease of 1.4-fold for HIV-1 X4 and 1.2-fold for HIV-1 R5 and an inhibition of 1.8-fold for HIV-1 X4 and 1.5-fold for HIV-R5, respectively. The influence of liposomes made with fractions 1, 3 and 7 was variable between replicates and virus tropism. TLC analyses (Figure S3) revealed the presence of PDIM and SL in fraction 2 and TDM in fractions 4 to 7 where TDM concentration decreased through fractionation. These results suggest that PDIM, SL and TDM are involved in disruption of DC-SIGN mediated HIV-1 *trans*-infection.

The role of SL1 was further investigated by generating liposomes from total lipids isolated from an H37Rv papA1Δ mutant (mutant missing the polyketide associated-protein-1, papA1, involved in biogenesis of SL1 and resulting in the absence of SL1 production) in parallel to liposomes with H37Rv papA1Δ total lipids complemented with soluble SL1 (Figure 6B). When we studied the impact of these liposomes on HIV-1 X4 (1) and HIV-1 R5 (2) *trans*-infection mediated by DC-SIGN, we observed that papA1Δ liposomes only decreased virus capture-transfer by 1.1-fold for HIV-1 X4 (*p*-value = 0.0053) and 1.2-fold for HIV-1 R5 (*p*-value = 0.0030) while H37Rv blocked *trans*-infection via DC-SIGN by 3.2-fold (*p*-value < 0.0001) for HIV-1 X4 and 3.1-fold (*p*-value < 0.0001) for HIV-1 R5. Interestingly, liposomes composed of papA1Δ total lipids complemented with SL1 were shown to cause strong inhibition of 3.4-fold for HIV-1 X4 (*p*-value < 0.0001) and 4.1-fold for HIV-1 R5 (*p*-value< 0.0001) *trans*-infection, indicating that SL1 expressed on H37Rv binds the DC-SIGN receptor, blocking HIV-1 *trans*-infection via this C-type lectin.

### 2.5. Differential Effects of Mtb Glycolipids on HIV-1 Trans-Infection Mediated via iDC and mDC

**Figure 7:**
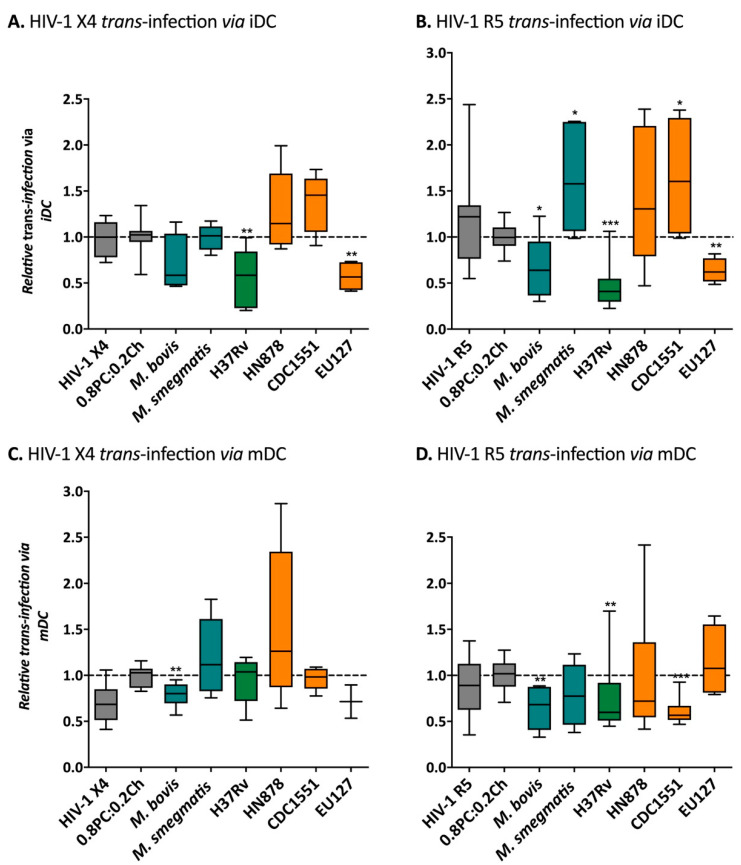
Influence of *Mycobacterium* liposomes on HIV-1 *trans*-infection via iDCs or mDCs. (**A**) and (**B**) iDCS, (**C**,**D**) mDCs, was pre-incubation for 30 min with 100 μg of liposomes or 50 µL of media. The cells were then incubated 2h with HIV-1 pseudo-typed virus: 12.5 ng CA-p24 pSG3-LAI (HIV-1 X4, panels (**A**,**C**)) or 20 ng CA-p24 pSG3-BAL (HIV-1 R5, panels (**B**,**D**)). For the data shown, the experiment has been performed using cells isolated from on at least two different donors where *n* ≥ 3 in total. The RLUs produced for each experiment were normalised to the average value of the negative control 0.8PC:0.2Ch liposomes. Mann–Whitney unpaired t-test was performed for (**A**–**D**) and *p*-value represented with * for *p*-value < 0.05, ** for *p*-value < 0.01, *** for *p*-value < 0.001, **** for *p*-value < 0.0001.

We next studied the impact of *Mycobacterium* liposomes on *trans*-infection of HIV-1 via DCs, the physiological relevant cell-types mediating HIV-1 capture-transfer. DCs were pre-incubated with liposomes before capture and transfer of HIV-1 X4 or R5 by DCs at different stages of maturation: iDCs (Figure 7A,B) and mDCs (Figure 7C,D). Interestingly, as observed with the Raji-DC-SIGN in vitro assays, HIV-1 X4 and R5 iDC mediated *trans*-infections were inhibited by 1.4- and 1.5-fold (*p*-value = 0.0450) in the presence of *M. bovis*; by 1.8- (*p*-value = 0.0025) and 2.1-fold (*p*-value = 0.0002) in the presence of H37Rv; and by 1.7- (*p*-value = 0.0011) and 1.6-fold (*p*-value = 0.0034) in the presence of EU127 liposomes, respectively. On the contrary, *M. smegmatis*, as well as *Mtb* HN878 and CDC1551 liposomes showed a trend toward increasing HIV-1 *trans*-infection via iDCs by 1.6-fold (HIV-1 R5); 1.3- (HIV-1 X4) and 1.5-fold (HIV-1 R5); and by 1.4- (HIV-1 X4) and 1.6-fold (HIV-1 R5, *p*-value = 0.0220) respectively (Figure 7A,B). These observations suggest that *M. bovis*, *Mtb* H37Rv and EU127 glycolipids are recognised by the DC-SIGN receptor expressed on iDCs causing inhibition of HIV-1 capture and transfer to target cells.

In parallel, iDCs were grown in the presence of poly(I:C) to allow maturation into mDCs and further used to mediate HIV-1 *trans*-infection to TZM-bl in the presence of *Mycobacterium* liposomes (Figure 7C,D). Although we observed variant efficiency of virus capture and transfer reflecting donor-to-donor variation, trends were observed when comparing the different mycobacterial strains. The presence of *M. bovis* liposomes prior to HIV-1 X4 and R5 capture-transfer via mDCs significantly inhibited the efficiency of *trans*-infection by 1.3- (*p*-value = 0.0071) and 1.5-fold (*p*-value = 0.0047) respectively. Similarly, H37Rv and CDC1551 liposomes blocked HIV-1 R5 viruses by 1.3- (*p*-value = 0.0052) and 1.6-fold (*p*-value = 0.0009) compared to the negative control 0.8PC:0.2Ch. For HN878, one mDCs donor did exhibit heightened transfer of HIV-1 X4 by 2.5-fold and which seemed to follow a trend of activation. Interestingly, cells from another donor showed an increase of HIV-1 X4 *trans*-infection in the presence of *M. smegmatis*. There is a clear variation in the impact of *Mycobacterium* liposomes on HIV-1 X4 and R5 *trans*-infection, depending on the stage of DC maturation and virus tropism. These results suggest that the DC-SIGN receptor is likely involved in HIV-1 DCs mediated *trans*-infection as well as other receptors expressed by iDCs and mDCs, explaining variations between the cells types used.

The above is supported when analyzing the effect of mannan, a DC-SIGN receptor antagonist, on HIV-1 *trans*-infection mediated by Raji-DC-SIGN, iDCs or mDCs (Figure S4). We tested two different concentrations of mannan, which was pre-incubated with each cell type prior to HIV-1 X4 *trans*-infection, as was performed for liposomes. We observed that Raji-DC-SIGN HIV-1 X4 *trans*-infection of TZM-bl cells is strongly inhibited with both concentrations of mannan tested with efficacy decreased to 0.16 with 20 µg/mL and in total with 2 mg/mL of mannan compared to virus alone. Blocking the DC-SIGN receptor with mannan on iDCs seems to not fully inhibit HIV-1 *trans*-infection. Indeed capture-transfer was partially blocked and displayed a dose dependent effect: 20 µg/mL mannan reduced efficacy by 1.5-fold and 2 mg/mL by 2.0-fold. Concerning capture-transfer via mDCs, we did not observe any inhibition to HIV-1 X4 capture-transfer but an increase was observed with 2 mg/mL by 1.6-fold. DC-SIGN recognition from antagonists such as mannan can decrease HIV-1 *trans*-infection via iDCs whereas on mDCs, the DC-SIGN antagonist could be seen to increase HIV-1 *trans*-infection at the highest concentration.

## 3. Discussion

Here we investigated the role of *Mycobacterium* lipid extracts presented via liposomes on influencing HIV-1 *cis*- and *trans*-infection. From this preliminary analysis of utilising generated liposomes containing lipid extract antigen from variant pathogenic (e.g., Mtb H37Rv, HN878, CDC1551 and EU127) and non-pathogenic (*M. bovis* and *M. smegmatis*) *Mycobacterium* strains we observed no difference between strains for influencing HIV-1 X4 or HIV-1 R5 direct infection of TZM-bl cells. The bacterial glycolipid antigens presented on liposomes therefore did not interfere with direct HIV-1 entry to target cells via either the CCR5 or CXCR4 co-receptor. This indicates that the interaction and binding of HIV-1 envelope protein sub-unit gp120 with CD4 receptor and co-receptors CCR5 and CXCR4 is not impacted by mycobacterial glycolipids. These results support that the mechanisms involved in enhancement of HIV-1 replication observed in co-infected patient [75,76] is most likely due to the modulation of the immune system by *Mtb* infection and do not involve an increase of HIV-1 entry efficacy via direct interaction with *Mtb* components.

However, our data suggest that *Mtb* glycolipids potentially impair or enhance HIV-1 *trans*-infection, where DCs capture and present virus to CD4 T cells, with important implications for virus transmission and dissemination. The capture of infectious virions and subsequent transmission to uninfected cells occurs by attachment of the glycoprotein Env to DC receptors such as MR or DC-SIGN [8,9,10,13,77,78]. We demonstrate that *trans*-infection of both R5 and X4 tropic HIV-1, mediated by DC-SIGN receptor, was impaired in the presence of *Mtb* glycolipids from *M. bovis*, *Mtb* H37Rv and *Mtb* EU127 strains. Unexpectedly, we identified TDM, SL1 and PDIM glycolipids to be involved in DC-SIGN recognition and impairment of HIV-1 *trans*-infection by using *Mtb* H37Rv total lipids fractionation. TDM is a major contributor of the immune simulation and is known to interact with C-type lectin receptor Mincle expressed on macrophages [37,79] but not DC-SIGN. Conversely, *Mtb* glycolipids PIMs, LM and LAM, identified to interact with C-type lectin receptors due to their mannoside caps [49,50,51], present in fraction 8 on H37Rv total lipids fractionation in our assays, did not show a clear impact on HIV-1 *trans*-infection mediated by DC-SIGN receptor. Concerning SL1, its role in *Mycobacterium* pathogenicity remains unclear [80,81]. In our model, we demonstrated using total lipids extract from the H37Rv mutant papA1Δ associated into liposomes, that SL1 is critical in the inhibition of DC-SIGN mediated HIV-1 *trans*-infection from *Mtb* H37Rv, suggesting SL1 interactions with DC-SIGN receptor. Additionally, the comparison between H37RvMA strains and H37RvAE differing from their origin and PDIM biosynthesis and composition [74], reveals the implication of PDIM in DC-SIGN/SL1 interactions. Indeed, the impairment of HIV-1 DC-SIGN mediated *trans*-infection with glycolipids from H37RvAE is lost using liposomes with total lipids extracted from H37RvMA. PDIM is known to be able to mask PAMPs to escape from macrophage recognition [45,46,48], suggesting that in our system, high levels of expression of PDIM in H37RvMA could mask SL1 interactions with DC-SIGN and not interfere with virus capture. Nevertheless, the interaction of PDIM, SL1 and TDM with DC-SIGN receptors remains to be characterised.

We tested the impact of the *Mtb* glycolipids associated into liposomes on HIV-1 *trans*-infection mediated by DCs at different stage of maturation. Our findings indicate that *M. bovis*, *Mtb* H37Rv and *Mtb* EU127 are able to inhibit HIV-1 *trans*-infection via iDCs. The similar observations obtained on HIV-1 capture/transfer mediated by DC-SIGN, suggests the involvement of DC-SIGN receptors in the inhibition of virus capture by iDC in the presence of glycolipids from these strains. Conversely, *M. smegmatis*, *Mtb* HN878 and *Mtb* CDC1551 liposomes showed a trend toward increasing HIV-1 *trans*-infection via iDCs. These results suggest the involvement of receptors other than DC-SIGN, such as MR, in *Mtb* antigen recognition, impacting HIV-1 *trans*-infection via mDC [10,11]. Moreover, the stage of DCs maturation influences HIV-1 *trans*-infection, where mDCs are more efficient at supporting virus *trans*-infection using different mechanisms compared to iDCs [19,82,83,84], glycolipids from *M. smegmatis*, *Mtb* HN878 and CDC1551 could activate iDCs maturation by pattern recognition receptors (PRRs) recognition and improve HIV-1 *trans*-infection efficacy. Interestingly, the impact of *Mtb* liposomes on mDCs mediated HIV-1 *trans*-infection varies from the experiments using iDCs. The variations observed between cell types supports the hypothesis that receptors other than DC-SIGN can impact HIV-1 capture and transfer and which can be differentially modulated by *Mtb* glycolipids. These complex interactions between bacterial antigens and immune cells will undoubtedly influence the ensuing immune responses generated. HIV-1 *trans*-infection mediated by mDCs, involved the receptor Siglec [16,17,18,19,20,84]. The influence reported here of *Mtb* liposomes on HIV-1 *trans*-infection mediated may be due to interactions with other PRRs, influencing Siglec expression, or direct interaction with Siglec.

Collectively, our findings indicate that variant strains of *Mtb* can provide differential effects on HIV-1 *trans*-infection which has the potential to influence HIV-1 disease course in co-infected individuals. Studying HIV-1 *trans*-infection mediated via DC-SIGN receptor represents a valuable alternative in vitro model to analyse DC-SIGN interactions with bacterial glycolipid components. Additionally, liposomes technology represents a useful technique to best mimic lipid distribution and antigen presentation on the mycobacterial cell wall. The activation of PRR signaling pathways by *Mtb* PAMPs leads to the activation of HIV-1 replication and infection [85] through various mechanisms including the modification/translocation of transcription factors [86,87], activation of NF-κB pathways, or cytokine production [88,89,90,91]. Liposome technology will provide a means to study the effects that *Mtb* antigens can exert on cell signaling and induction of immune responses impacting HIV-1 infection, and more generally, the effect of bacterial antigens on the immune responses.

## 4. Materials and Methods

### 4.1. Commercial Lipids

Cholesterol (Ch) and phosphatidylcholine (PC) were purchased from Sigma-Aldrich (Sigma-Aldrich, St. Louis, MO, USA), and SL1 lipid solutions from BEI Resources.

### 4.2. M. Tuberculosis Strains and H37Rv Mutants

*Mtb* H37RvAE was used for all experiments unless otherwise mentioned. The EU127 *Mtb* strain was selected from a biobank of *Mtb* strains collected in a clinical study conducted at the uBuntu clinic in Site B, Khayelitsha, a peri-urban township 30 km outside of Cape Town, South Africa. Spoligotyping of this indicated that EU127 was a Beijing genotype. The HN878, CDC1551 and H37RvMA *Mtb* isolates were propagated as part of the same strain library and included as controls. H37RvAE Δ papA1 was constructed by specialized transduction as described [92].

*Mtb* glycerol stocks were inoculated into 10 mL of 7H9 (BD, Franklin Lakes, NJ, USA) media with ADC (BD, Franklin Lakes, NJ, USA) and 0.05% Tween 80 (Sigma-Aldrich, St. Louis, MO, USA) supplementation and cultured at 37° for 10 days, with gentle agitation every two days. On day 10, 1 mL of this culture was used to inoculate 100 mL of 7H9 media with ADC supplementation (without Tween 80) and cultured for a further 10 days at 37 °C without shaking. Cultures were centrifuged at 8000× rpm for 10 min to pellet the cells. The supernatant was removed and discarded, and the pellet was resuspended in 1 mL of PBS. *Mtb* was killed by boiling cell suspensions at 80 °C for 1 h and used from lipid extractions and preparation of liposomes.

### 4.3. Lipids Extractions

Total lipid extracts were obtained from culture pellet of *M. bovis* (BCG), *M. smegmatis* (MC^2^155), *Mtb* H37Rv (H37RvAE), H37RvMA, H37RvAE Δ papA1, HN878, CDC1551 and EU127 following standard procedures as described [93]. Shortly, a solution of CHCl_3_:MeOH:H_2_O was added to the bacterial pellet and kept at 50 °C for 3h. After centrifugation at 3000× *g* rpm, a mix of CHCl_3_:H_2_O was added to the supernatant and centrifuged after mixing. The bottom phase obtained was then washed 2× *g* by with a mix of CHCl_3_:MeOH:H_2_O (3:47:48 proportions) and heat at 55 °C to obtained a dry pellet.

For H37Rv (H37RvAE) lipids fractionation, 2 mg of total lipid extract from *Mtb* H37RvAE was fractioned using a silica column. The column was pre-washed with chloroform (3× void volume) before the sample was added. Once the input sample was added onto the column, 100 mL of chloroform was added to start flow through collection. A mix of CHCl_3_:MeOH solvent was passed through the column with a progressive increase in the proportion of methanol (99:1, 1–8 collection tubes; 98:2, 9–15 collection tubes; 97:3, 16–23 collection tubes; 95:5, 24–31 collection tubes; 93:7, 32–49 collection tubes; 90:10, 50–57 collection tubes and 80:20, 58–63 collection tubes). For the analyses of H37RvAE, collection tubes 1 to 9 were pooled as Fraction 1; 10 to 13 pooled as Fraction 2; 14 to 18 pooled as Fraction 3; 19 to 27 pooled as Fraction 4; 28 to 32 pooled as Fraction 5; 33 to 36 pooled as Fraction 6, 37 to 44 pooled as Fraction 7 and 45 to 63 pooled as Fraction 8.

### 4.4. Liposomes

All dried lipid pellets were solubilised in CHCl_3_:MeOH (2:1 proportion) and PC and Ch were solubilised in chloroform. Liposomes were generated with a mix of PC and Ch in 0.8PC:0.2Ch proportion for liposomes without *Mycobacterium* lipids; a mix of PC, Ch and total lipids extracted from BCG, MC^2^155, H37RvAE, H37RvMA, H37Rv papA1Δ, H37RvAE Fractions, HN878, CDC1551 and EU127 in 0.6PC:0.2Ch:0.2 *Mycobacterium* proportion; a mix of PC, Ch, H37Rv papA1Δ and SL1 lipids in 0.6PC:0.2Ch:1.9:0.1SL1; or a mix of PC, Ch and PDIM lipids in 7.8PC:0.2Ch:0.2PDIM proportion. After the different lipids were mixed together in chloroform solution (2 mg in total), the chloroform was then evaporated with nitrogen gas for 30 min forming a film of dried lipids. Liposomes were hydrated by addition of Roswell Park Memorial Institute (RPMI-1640, Thermo Fisher Scientific, Waltham, MA, USA), Dulbecco Modified Eagle Medium (DMEM, Thermo Fisher Scientific, Waltham, MA, USA), Phosphate-Buffered Saline (PBS, Thermo Fisher Scientific, Waltham, MA, USA), or sterile water. Liposomes were then incubated at 55 °C for 30 min with vortexing (final concentration 10 mg/mL). The preparations were subsequently sonicated for 30 min at 4 °C.

For NanoSight analyses of liposomes, 0.8PC:0.2Ch liposomes were filtered using the Avanti^®^ Mini-Extruder system after sonication. Liposome solutions were filtered through a 100 or 200 nm polycarbonate filter under 50 °C temperature to produce 100 and 200 nm 0.8PC:0.2Ch liposome diameters.

### 4.5. Liposomes Characterisation

For TLC analyses, 10 µL of 0.8PC:0.2Ch, BCG and H37RvAE liposomes made in sterile water, SL, TDM, PIMs, PDIM lipid solution or H37RvAE lipid Fractions 1 to 8 were spotted and dried on a silica gel 60 F254 plate (Sigma-Aldrich, St. Louis, MO, USA). Sample separation occurred in 60:16:2 CHCl_3_:MeOH:H_2_O solvent and was visualised by staining with molybdophosphoric acid (MPA) and charring.

Visualisation, particle concentration and the size distribution of the generated liposomes were evaluated using the NanoSight NS300 instrument (Malvern Panatical, Malvern, UK) and using Nanoparticle Tracking Analysis (NTA) software. Videos were recording at camera level 13. The post-acquisition settings were with a minimum detection threshold 7, automatic blur and automatic minimum expected particle size. 0.8PC:0.2Ch, BCG and H37RvAE liposome made in PBS were diluted 1:1000–1:2000 in PBS for each sample and three 60 s videos were recorded and analysed.

### 4.6. Cells

293T and TZM-bl (NIH AIDS) cells were maintained at 37 °C 5% CO_2_ in DMEM supplemented with 10% FBS, L-glutamine, 100 U/mL penicillin and 100 mg/mL streptomycin. Raji-DC-SIGN (NIH AIDS) were cultured at 37 °C 5% CO_2_ in RPMI-1640 containing l-glutamine, supplemented with 10% FBS, 100 U/mL penicillin and 100 mg/mL streptomycin.

Human blood monocytes were isolated in buffy coats from healthy donors by using Ficoll gradient and a subsequent CD14 selection step using the MACS system (Miltenyi Biotec, Bergisch Gladbach, Germany). Purified monocytes were cultured RPMI-1640 containing L-glutamine supplemented with 10% FBS serum 100 U/mL penicillin, 100 mg/mL streptomycin, 70 ng/mL human IL-4 (Thermo Fisher Scientific, Waltham, MA, USA) and 50 ng/mL human GM-CSF (Thermo Fisher Scientific, Waltham, MA, USA) for differentiation into immature dendritic cells (iDC). The iDCs were harvested after 6 days of incubation. Mature monocyte derived dendritic cells (mDCs) were isolated from fresh iDCs. At day 620 µg/mL of Poly(I:C) (Sigma-Aldrich, St. Louis, MO, USA) was added to iDCs media. The mDCs cells were harvested after 18–24 h of incubation at 37 °C, 5% CO_2_.

### 4.7. Cell Viability in the Presence of Liposomes

3 × 10^4^ TZM-bl cells in 250 μL media per well were seeded in 96-well plates. After 24 h at 37 °C 5% CO_2_, the cells were incubated with 1000, 100, 10 and 1 ng of 0.8PC:0.2Ch liposomes in 250 μL total volume. After 48 h incubation at 37 °C 5% CO_2_ the media was removed and the cells harvested via trypsin treatment and fixed in 2% PFA (Sigma-Aldrich, St. Louis, MO, USA). After fixation, the cells were re-suspended in PBS and the viability was analysed by standard flow cytometry measuring forward and side scatter to determine live from dead cell populations. BD Accuri^TM^ C6 was used to record 10,000 events for each sample and data analysis was performed using the BD Accuri^TM^ C6 Plus software. Furthermore, no differences in cell recoveries were observed following culturing in the presence of the variant concentrations of liposomes.

### 4.8. Virus Production

One day prior to transfection 1.2 × 10^6^ 293T cells were seeded in a 10 cm dish in 8 mL of media. After 24 h, the cells were transfected using the Lipofectamine standard method where 2 μg of HIV-1 envelope plasmid BAL (R5 tropism, NIH AIDS) or LAI (X4 tropism, NIH AIDS), and 2 μg of the backbone pSG3*Δenv* plasmid (NIH AIDS) were mixed in 300 μL of OptiMEM (Thermo Fisher Scientific, Waltham, MA, USA). Separately, 24 μL of Lipofectamine was mixed with 276 μL of OptiMEM and 300 μL of this was added to the DNA mix. After 30 min incubation at RT, the Lipofectamine/DNA mixture was added dropwise to 293T cells with 1 mL of OptiMEM. After 6h, the media was replaced with fresh DMEM and the pseudo-typed virus stock (supernatant) was harvested after further 48 h of incubation at 37 °C 5% CO_2_. Viruses were stored at −80 °C and virus concentrations were quantified by measuring CA-p24 antigen levels by ELISA (Aalto Bio Reagents, Dublin, Ireland).

### 4.9. HIV-1 Cis-Infection

One day prior to infection, 3 × 10^4^ TZM-bl cells in 250 μL of media per well were seeded in 96-well plates. In scenario one, liposomes suspension in DMEM was added to the cells at the same time virus was added. 8ng CA-p24 of pSG3-LAI, pSG3-BAL pseudo-typed virus was mixed with 100 ng liposomes in 50 μL total volume and added to the TZM-bl cells. After 2 h incubation at 37 °C 5% CO_2_, 200 μL of media was added and the infection measured after 48 h. In the second scenario, liposome suspensions in DMEM were added to the cells prior to virus input. 50 μL of 100ng of liposome was added to the TZM-bl cells. After 30 min incubation at 37 °C and 5% CO_2_, 8 ng CA-p24 of pSG3-LAI or pSG3-BAL pseudo-typed virus was added and incubated for 2 h as described above. Following this incubation, 200 μL of media was added to each well and incubated for 48 h. For both scenarios, after 48 h of infection the cells were washed with PBS, and 20 μL of lysis Buffer (Promega, Madison, WI, USA) was added per well. Luciferase activity was determined using the Luciferase Assay kit (Promega, Madison, WI, USA) and FLUOStar Omega luminometer (BMG LabTech, Ortenberg, Germany) following the manufacturer’s instructions. Infections with pSG3Δenv virus were used as negative control.

### 4.10. HIV-1 Trans-Infection

One day prior to capture/transfer, 3 × 10^4^ TZM-bl cells per well were seeded on 96-well plates and cultured overnight. Prior virus capture, 0.5 × 10^6^ Raji DC-SIGN, iDCs or mDCs cells were pelleted and mixed with 100 μg of liposomes in RPMI-1640 suspension. After 30 min incubation at 37 °C 5% CO_2_, 12.5 ng CA-p24 pSG3Δenv, 12.5 ng CA-p24 pSG3-LAI or 25 ng CA-p24 of pSG3-BAL were added to cells for 2 h at 37 °C 5% CO_2_. The cells were then washed 4× with cold PBS and re-suspended in 45 μL of DMEM containing 30 ng/mL of Dextran. Finally, 15 μL of the suspension was used per well to infect TZM-bl cells. After 48 h at 37 °C 5% CO_2_, the TZM-bl cells were lysed and luciferase activity measured as described above. Infection with pSG3Δenv virus were used as negative control.

### 4.11. Statistical Analysis

For all results, unpaired sample comparisons were performed using the non-parametric rank test Mann–Whitney and depict in Figures using the following: * for *p*-value < 0.05, ** for *p*-value < 0.01, *** for *p*-value < 0.001, **** for *p*-value < 0.0001. Data are represented in boxplot where the box extends from the 25th and 75th percentiles, the median is plotted at the line in the middle of the box, the whiskers represent the minimum and maximum value. All statistical analyses were performed with the Prism software.

## Data Availability

Data available on request.

## Supplementary Materials

The following are available online at https://www.mdpi.com/1422-0067/22/4/1945/s1.

## Abbreviations

AG	Arabinogalactan
BCG	Bacille Calmette Guerin
CCR5	CC chemokine receptor 5
Ch	Cholesterol
CXCR4	CXC chemokine receptor 4
DC	Dendritic cell
DC-SIGN	Dendritic cell-specific ICAM-3-grabbing non-integrin
HIV-1	Human Immunodeficiency Virus type 1
iDC	Immature dendritic cell
LAM	lipoarabinomannan
LM	lipomannan
LTR	Long Terminal Repeat
MA	Mycolic acids
Mincle	macrophage-inducible C-type lectin
mDC	Mature dendritic cell
MR	Mannose receptor
*Mtb*	*Mycobacterium tuberculosis*
PAMPs	Pathogen associated molecular patterns
papA1	Polyketide associated-protein-1
PC	Phosphatidylcholine
PDIM	Phthiocerol dimycocerosate
PG	Peptidoglycan
PGL	Phenolic glycolipids
PIM	Phosphatidylinositol mannoside
PRR	Pattern recognition receptors
SL	Sulfolipid
TB	Tuberculosis
TDM	Trehalose dimycolate
TLC	Thin-layer chromatography
