# Supplementary material for: Generation of Liposomes to Study the Effect of Mycobacterium Tuberculosis Lipids on HIV-1 cis- and trans-Infections
Source: Int J Mol Sci. 2021 Feb 16;22(4):1945. doi: 10.3390/ijms22041945 (PMC7920488; doi:10.3390/ijms22041945)
Supplement: Supplementary file 1 [file ijms-22-01945-s001.pdf]

International Journal of
Molecular Sciences

Supplementary Figures

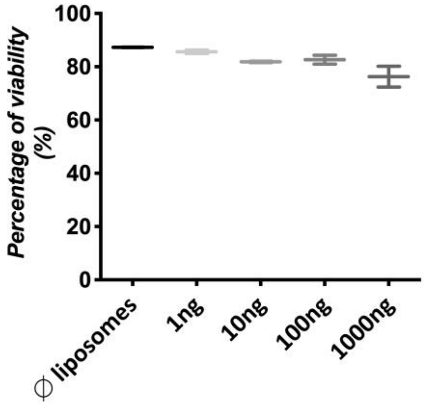

Figure S1. Viability of TZM-bl with 0.8PC:0.2Ch liposomes. $3x10^4$ TZM-bl cells per well were seeded in 96-well plates. After 24h at 37°C 5% CO2, the cells were incubated with 1000ng, 100ng, 10ng and 1ng of 0.8PC:0.2Ch liposomes in 250µl total volume. After 48h incubation at 37°C 5% CO2 the media was removed and the cells harvested via trypsin treatment and fixed in 2% PFA (Sigma Aldrich, UK). After fixation, the cells were re-suspended in PBS and the viability was analysed by flow cytometry. BD Accuri™ C6 was used to record 10,000 events for each sample and data analysis was performed using the BD Accuri™ C6 Plus software. For the data shown, n=2. Mann Whitney unpaired t-test was performed.

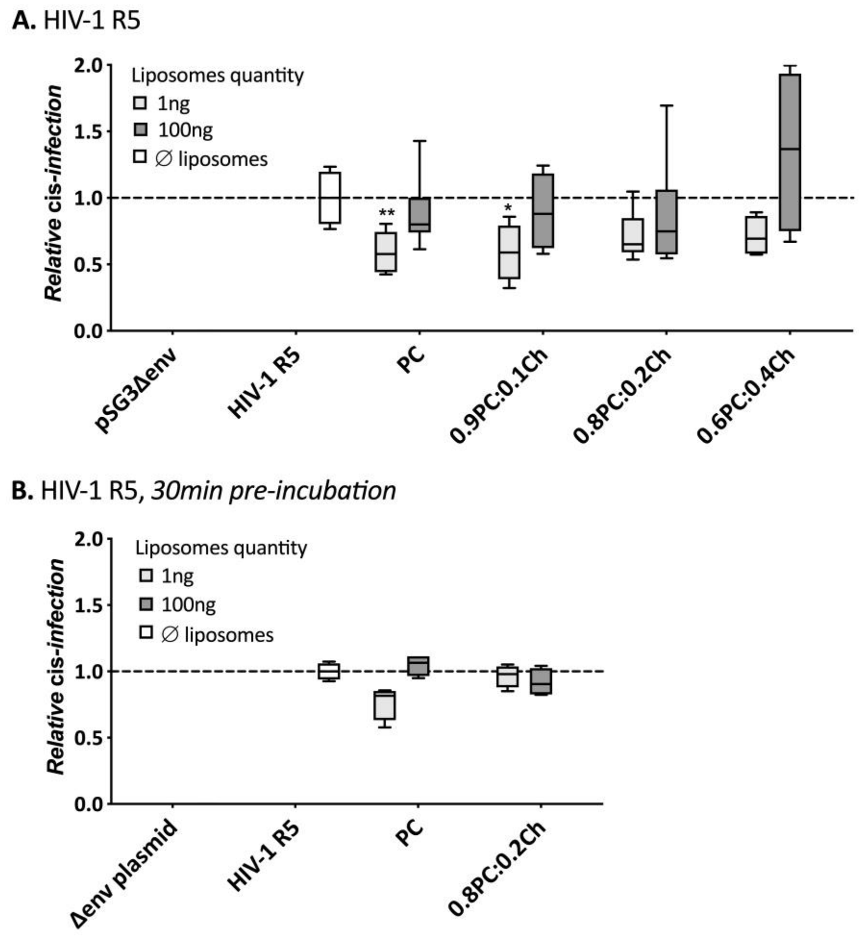

Figure S2. : Influence of PC and Ch in liposome composition on HIV-1 *cis*-infection. $3x10^4$ TZM-bl cells per well were seeded in 96-well plates. After 24h, the cells were infected with 8ng CA-p24 of pSG3-JF-FL (HIV-1 R5) and pSG3Δenv (ΔpSG3) where (**A**) virus input with 1 or 100ng of liposomes at the same time or (**B**) 1 or 100ng of liposomes added to TZM-bl cells 30min prior to adding virus. Liposomes tested: PC, 0.9PC:0.1Ch, 0.8PC:0.2Ch and 0.6PC:0.4Ch. 48h post-infection cells were lysed and luciferase activity measured (RLU). RLU produced on each experiments were normalised to the average value of the negative control ΔpSG3. For the data shown, n=4. Mann Whitney unpaired t-test was performed P value represented with * for P value< 0.05, ** for P value< 0.01, *** for P value< 0.001, **** for P value< 0.0001.

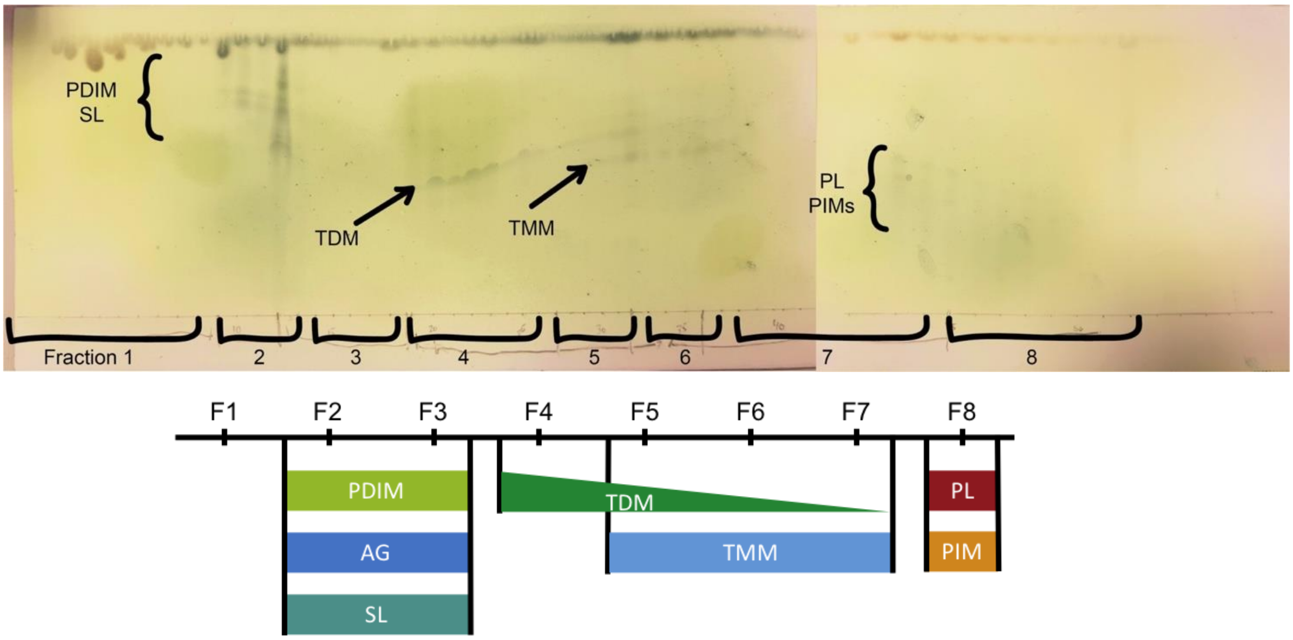

Figure S3. Thin-layer chromatography analyses of H37Rv Fractions. TLC of H37Rv's fractions occurred from H37RvAE total lipid extract in 60:16:2 CHCl3:MeOH:H2O solvent and visualised by staining with MPA and charring. Fractions (1-9) were pooled as fraction 1, (10-13) pooled as fraction 2, (14-18) pooled as fraction 3, (19-27) pooled as fraction 4, (28-32) pooled as fraction 5, (33-36) pooled as fraction 6, (37-44) pooled as fraction 7, and (45-63) pooled as fraction 8. The data shown are from one representative experiment.

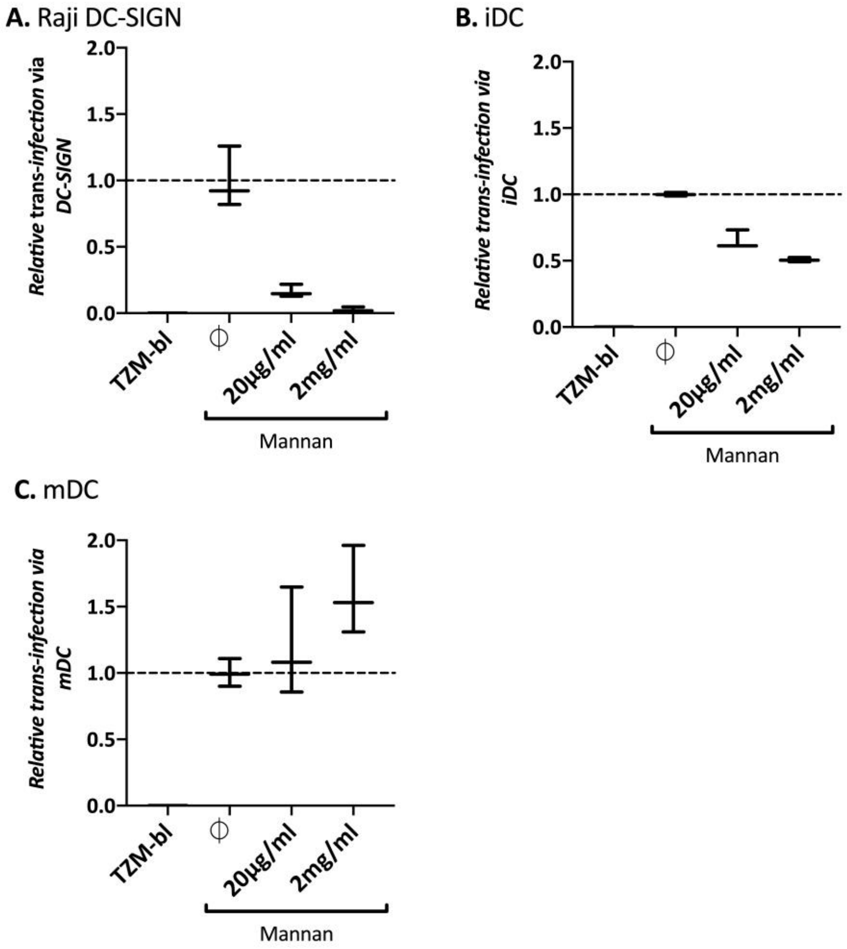

Figure S4. HIV-1 *trans*-infection on TZM-bl cells in presence of mannan. 0.5x10[6] Raji-DC-SIGN(**A**), iDCs (**B**) and mDCs (**C**) were pre-incubation for 30min with 20μg/ml, 2mg/ml of mannan or 50μl of media (∅). The cells were then incubated for 2h with 12.5ng CA-p24 pSG3-LAI HIV-1 X4 pseudo-typed viral particles. After capture the cells were washed and co-cultured with TZM-bl cells. The luciferase activity was read after 48h. RLU produced on each experiments were normalised to the average value of the negative control HIV-1 X4 *trans*-infection without mannan. The data shown are representative of one experiment using cells isolated from one donor with n=3. Mann Whitney unpaired t-test was performed.
